# Relocation of chloroplast proteins from cytosols into chloroplasts

**DOI:** 10.1080/15592324.2023.2258321

**Published:** 2023-09-14

**Authors:** Kwanuk Lee

**Affiliations:** Department of Biology, Jeju National University, Jeju, Korea

**Keywords:** chloroplast-artificial targeting system, chloroplast proteins, chloroplast genome, transformation

## Abstract

The chloroplasts in terrestrial plants play a functional role as a major sensor for perceiving physiological changes under normal and stressful conditions. Despite the fact that the plant chloroplast genome encodes around 120 genes, which are mainly essential for photosynthesis and chloroplast biogenesis, the functional roles of the genes remain to be determined in plant’s response to environmental stresses. Photosynthetic electron transfer D (PETD) is a key component of the chloroplast cytochrome b_6_f complex. Chloroplast ndhA (NADH dehydrogenase A) and ndhB (NADH dehydrogenase B) interact with photosystem I (PSI), forming NDH-PSI supercomplex. Notably, artificial targeting of chloroplasts-encoded proteins, PETD, NDHA, or NDHB, was successfully relocated from cytosols into chloroplasts. The result suggests that artificial targeting of proteins to chloroplasts is potentially open to the possibility of chloroplast biotechnology in engineering of plant tolerance against biotic and abiotic stresses.

Chloroplasts are powerhouses that harbor photosynthetic machinery. The organellar genome size is approximately 120–190 kb and is determined as circular and double-stranded DNA structures.^1^ The chloroplast genome of land plants encodes approximately 100–120 genes, which consist of key proteins indispensable for photosynthetic machinery, ATP production, and cellular metabolic pathway. The plant chloroplasts play an essential role as a sensor of environmental stresses including high light, high and low temperature, flooding, drought, and high salt.^2–4^ The change of photosystem complex in plant’s response to environmental cues influences photosynthetic electron chain, thereby resulting in the alteration of carbohydrate biosynthesis and Calvin–Benson cycle. Moreover, chloroplasts with these physiological changes transmit a signal to the nucleus, known as chloroplast-to-nucleus retrograde signaling, to maintain chloroplast homeostasis by remodeling of nuclear gene expression, protein, and metabolites, which ultimately confer proper adaptation to the environmental stresses.^5–8^

The chloroplasts have been utilized as an attractive means to enhance photosynthetic capacities and yields in plants, as well as to produce vaccines, pesticides, and enzymes via synthetic biology.^9,10^ Interestingly, transplastomic plants represent chloroplast transformations in which target genes are integrated into chloroplast genome. This would be a good tool for artificial delivery of target genes and protein expression in host chloroplasts.^9,11^ Since a plant contains a large number of chloroplasts, an enormous amount of target proteins can be obtainable. However, the biotechnology still exhibits some barriers that are applicable to only a few species including tobacco and Arabidopsis as well as a different posttranslational modifications in chloroplasts.^10–12^ Instead, strategies for transient gene expression or transgenic lines, in which chloroplast genes are translated in the cytosols and targeted to the chloroplasts, can be utilized in a variety of plants.

Mitochondrial alternative oxidase 1a whose own mTP was replaced with the cTP of rubisco small subunit (RbcS) was successfully localized to chloroplasts and restored a plastid terminal oxidase activity in the *immutans* mutants of Arabidopsis.^13^ A transformation of the cTP-chloroplast ribosomal protein S12 (rps12) fusion into the nuclear genome rescued the Arabidopsis *ppr4* mutant that is impaired in the correct splicing of chloroplast *rps12* intron 1b.^14^ Similarly, mitochondrial NADH dehydrogenase subunit 7 (NAD7) fused with the mTP of Arabidopsis F1-ATPase γ-subunit (pFAγ) restored the phenotype of slow growth 3 mutant that is defective in the splicing of mitochondrial *nad7* intron 2.^15^ Moreover, artificial chloroplast targeting of phytochelatin synthase enhanced the tolerance of Arabidopsis against heavy metal stress.^16^ Cholesterol oxidase and *cry1Ac* were successfully targeted to tobacco chloroplasts^17^ or rice^18^ and chickpea chloroplasts,^19^ leading to an improved resistance against larvae and insects, respectively. These observations indicate that artificial trafficking system to organelles can enhance photosynthetic efficiency and plant’s tolerance against abiotic and biotic stresses.

Chloroplast gene expression is influenced by abiotic stresses. For instance, chloroplast photosynthetic electron transfer D (PETD) constitutes a subunit of the chloroplast cytochrome b_6_f complex, which is essential for electron flow.^20^ Notably, half of chloroplast PETD was reduced in plant’s response to drought stress.^21^ The chloroplast ndhA (NADH dehydrogenase A) and ndhB (NADH dehydrogenase B) are associated with photosystem I (PSI), which forms the NDH-PSI supercomplex.^22^ Chloroplast ndhA and/or ndhB expression declined under low temperature, high light, and humidity stress,^23–27^ indicating that the NDH complex plays a crucial role as a safeguard of the photosynthetic machinery against environmental stresses.

First, to generate the vector harboring transit peptides that enable cytosolic proteins to target into chloroplasts, the 59-amino acid peptide derived from RbcS, including 24-amino acid of chloroplast transit peptide (cTP) in Arabidopsis,^28^ was utilized. The cTP was fused in frame with the coding sequences of PETD, NDHA, and NDHB in the cloning vector, pET22a (+) using the restriction enzymes, BamH I and Xho I. The cTP-target constructs were inserted into the expression vector of green fluorescent protein (GFP), CsV-GFP, using the restriction enzymes, Xba I and BamH I. The target proteins were expressed under the control of a *Cauliflower mosaic virus* 35S promoter (Figure 1a). The Agrobacterium strain GV3101 containing each construct of 35:cTP-PETD-GFP, 35:cTP-NDHA-GFP, or 35:cTP-NDHB-GFP was co-infiltrated with the P19 suppressor of viral gene silencing into 3-week-old tobacco (*Nicotiana benthamiana*) leaves.^29^ The plants were further grown in the room temperature at 23°C for 3 d under long-day conditions (16 h light/8 h dark period). The subcellular localization was investigated using confocal microscope. The results revealed that significant GFP signals of expressing PETD, NDHA, and NDHB were detected mainly in chloroplasts (Figure 1b).

**Figure 1.**
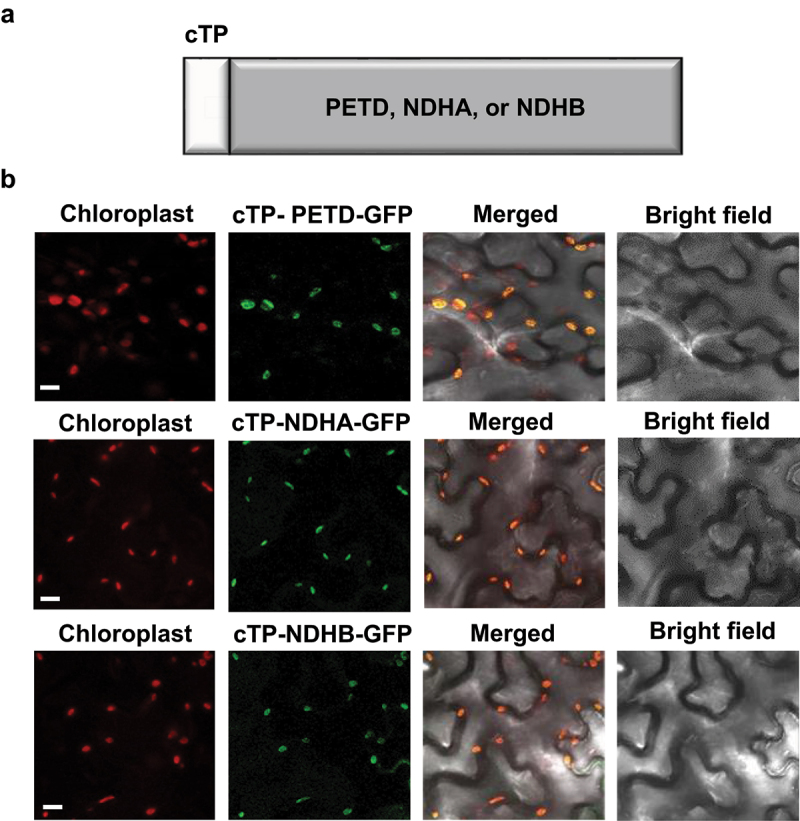
The domain structure and subcellular localization of PETD, NDHA, and NDHB. (a) schematic representation of the PETD, NDHA, and NDHB proteins for artificial targeting to chloroplasts. Chloroplast transit peptide (cTP) was fused in front of the PETD, NDHA, or NDHB genes. (b) Subcellular localization of the cTP-PETD, NDHA, or NDHB-GFP proteins. The plants of *N. benthamiana* were grown in a mixture of vermiculite, peat moss, and perlite at 23 ± 2°C under long-day (LD) conditions (16 h light/8 h dark cycle). GFP signals from transiently expressing cTP-PETD, NDHA, or NDHB in 3-week-old *N. benthamiana* leaves were observed via a confocal microscope. The excitation and emission wavelength was 488 nm and 545 nm for GFP and 635 nm and 680 nm for chlorophyll autofluorescence, respectively. The red signal and the green signal indicate chlorophyll autofluorescence and GFP fluorescence in chloroplasts, respectively. Bar, 10 μm.

These results suggest that transient expression of chloroplasts proteins, PETD, NDHA, and NDHB, can be successfully relocated from the cytosols into the chloroplasts. Chloroplast PETD, NDHA, or NDHB are important for proper functioning of photosystems in plant’s response to optimal and stressful conditions.^23–27^ Given that the chloroplast gene expression is altered by environmental stresses, overexpression of the chloroplast proteins could promote the photosynthetic rate in chloroplasts. The ectopic expression could further facilitate the plant’s tolerance against the harmful stresses, thereby resulting in enhanced growth and yields (Figure 2). Artificial targeting of PETD, NDHA, and NDHB, which are overexpressed in chloroplasts, should be assessed in stable transgenic plants. The functional analysis of chloroplast genes by the approaches of artificial targeting and the transplastomic technology together with reverse genetics will confer a profound understanding of the evolution of chloroplast genome across diverse organisms. In addition, this will shed light on novel chloroplast biotechnology in engineering of plant resistance and tolerance against biotic and abiotic stresses.

**Figure 2.**
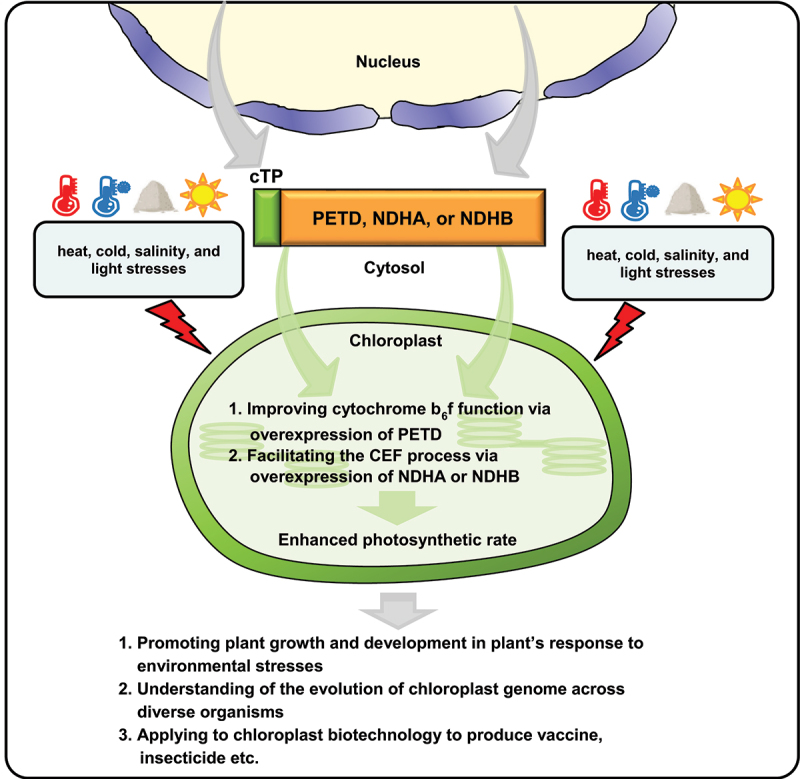
Relocation of chloroplast PETD, NDHA, and NDHB proteins from cytosols to chloroplasts. Artificial targeting of chloroplast PETD, NDHA or NDHB can contribute to improving the function of the cytochrome b_6_f complex as well as facilitating the cyclic electron flow (CEF), thus possibly conferring enhanced photosynthesis efficiency in plant’s response to abiotic stresses including heat, cold, salinity, and light stresses. In addition, the artificial targeting techniques can contribute to understanding the evolution of chloroplast genomes and can be applied to chloroplast biotechnology to produce vaccines and insecticides against biotic stresses.
